# MicroRNA loaded edible nanoparticles: an emerging personalized therapeutic approach for the treatment of obesity and metabolic disorders

**DOI:** 10.7150/thno.71399

**Published:** 2022-03-05

**Authors:** F. Campolo, G. Catanzaro, M.A. Venneri, E. Ferretti, Z.M. Besharat

**Affiliations:** Department of Experimental Medicine, Sapienza University, 00161 Rome, Italy.

**Keywords:** edible nanoparticles, microRNA, exosomes, obesity, personalized medicine

## Abstract

Obesity is a metabolic chronic disease whose prevalence is strongly growing in the last years, reaching pandemic proportions. Nowadays weight loss, achieved through lifestyle changes, is the first line therapeutic objective, although great inter-individual variabilities influence response to treatment, suggesting the involvement of epigenetic factors. In this contest, there is increasing recognition of the role of small RNA molecules, particularly microRNAs in the epigenetic regulation of genes involved in adipose tissue and glucose metabolism and several microRNAs have been found to be dysregulated in obesity and metabolic diseases. The development of novel personalized therapeutic strategies using microRNAs bears promise. However, the application of naked microRNAs has been hampered by their low specificity and sensitivity. In a recent issue of *Theranostics*, Kumar et al. explored the possibility of microRNA delivery through ginger-derived nanoparticles (GDNPs) as an alternative therapeutic approach for obesity treatment.

The results reported by Kumar et al., addressing non-coding RNAs and edible plant derived nanoparticles, open new perspectives for the application of this innovative and safe delivery system in the clinical practice for the treatment of obesity and other metabolic disorders.

Obesity, defined as abnormal or excessive fat accumulation, represents a health risk and is a major public health concern that has underwent a marked increase over the past four decades 1. It represents a complex and multifactorial health issue resulting from a combination of behavioral and genetic factors 2 with excess of body weight, mostly due to high-fat diet, as one of the most important risk factors 3. It is estimated that there are more overweight/obese than underweight adults making this pathology an emergency of pandemic proportions 4. The growing incidence, together with the presence of comorbidities, such as cardiovascular and chronic liver diseases, has strongly attracted worldwide attention to develop new effective therapeutical approaches against obesity and related disorders. Obesity first line treatment is represented by lifestyle modification to promote weight-loss, but in most cases this approach is insufficient and a pharmacological treatment and/or surgical interventions are needed 5. Although more than 10.000 clinical trials focusing on obesity have been registered worldwide 6, few drugs for obesity treatment are currently available, mostly because of their side effects. This gap presents an urgent need for more effective therapeutical approaches in the treatment or prevention of obesity and related disorders.

The desired strategy should ideally combine an increased therapeutic efficacy with reduced systemic side-effects. Small non-coding RNAs have been demonstrated to carry strong therapeutic potential for obesity and metabolic syndromes 7 and several research groups have investigated the expression of microRNAs in adipose tissue between lean and obese subjects 8. Therefore microRNAs, as part of a therapeutic strategy, hold promise, although their delivery continues to be a major challenge for this research field. In fact, the *in-vivo* efficiency of naked microRNAs, both in terms of distribution, stability and therapeutic index, is low. Thus, to increase their molecular stability, microRNAs should be encapsulated to obtain benefits in terms of both therapeutic efficacy and systemic side effects reduction 9. In the last years, a number of innovative nanotechnological methods have been implemented and some are currently under development. Among the different methods, exosomes present the most natural delivery system since they have been observed as a cell-to-cell communication method in the human body. However, there are many concerns related to exosomes-delivery systems, isolation techniques, safety and efficacy after surface engineering 10. Other “man-made” nanodelivery systems may be based on different formulations, even though synthetic nanoparticle-based drug delivery systems show relatively high immunotoxicity and high costs as compared to traditional drug formulations. Conversely, plant-derived nanoparticles do not present the above-mentioned limitations; they are naturally occurring biocompatible vesicles that do not generate immunotoxicity and are easy to obtain. In this context, the Ginger derived nanoparticles (GDNPs) that Kumar et al. 11 described in *Theranostics* have tissue specific targeting properties, showing great stability in the gastrointestinal system, beyond presenting colon-targeted delivery and high intestinal epithelium permeability. It has been demonstrated that GDNPs protect mice from alcohol-induced liver damage 12, inflammatory bowel disease 13 and present high anti-inflammatory efficacy in a mouse model of colitis 14. The novel finding of Kumar et al. is the effect of ginger derived nanoparticles (GDNP) on gut epithelial aryl hydrocarbon receptor (AhR) in a mouse model of high fat diet (HFD)-induced type 2 diabetes mellitus (T2DM) 11.

The theoretical foundations of this work rely on the recognized role of AhR in the regulation of glucose metabolism 15 through a fine integration of environmental, microbial and metabolic cues 16 and the known HFD-induced obesity protective effect of ginger 17. To understand the molecular effectors and signaling pathway governing the association “diet - AhR - GDNP - insulin pathway” the authors performed a deep analysis of microRNAs enriched exosomes isolated from HFD mice treated with GDNP. They focused their attention on miR-375, an efficacious regulator of β cells glucose metabolism and insulin secretion 18. MiR-375 expression levels were induced after GDNP treatment *in vitro* and *in vivo* leading to the investigation of its putative targets.

Among them, AhR 3'UTR displayed a strong potential target site for miR-375 leading authors to test whether miR-375 regulated AhR. To this end, nano-miR-375 packaged GDNP were administered to wild-type mice demonstrating, without any reasonable doubt, that miR-375 inhibits the expression of AhR in a GDNP dose-dependent way. This direct relationship is rapidly revised by the presence of a third player, VAMP7, a vesicle-associated membrane protein involved in the biogenesis and secretion of exosomes 19, obtained by the pivotal microarray analysis of genes modulated by GDNP treatment. Through a large series of complex and exhaustive experiments Kumar and co-workers finally demonstrated that VAMP7 induction led to the increase of exosomal miR-375 expression in the presence of GDNP, configuring VAMP7 as a tight regulator of miR-375 intracellular levels. Additionally, when high doses of GDNP and consequently high levels of VAMP7 were present, miR-375 was sorted into fecal exosomes to prevent insulin resistance mediated by bacterial indole and hepatic AhR.

In conclusion, the work presented by Kumar and colleagues showed the authors' clear understanding of nanotechnology and microRNA-packaged nanoparticles characterization in *in vitro* and *in vivo* models, which is highly desired in the pursuit of innovative target therapies for metabolic diseases.

Taken together, these results provide emerging evidence for the development of edible nanoparticles as a safe delivery system for microRNAs targeting different tissues, either by systemic or oral administration, as therapeutic agents which could represent a significant advancement in the treatment of metabolic diseases (**Figure 1**).

Nevertheless, some aspects need to be taken into consideration to develop personalized treatments using microRNA loaded edible nanoparticles. Scientists need to address delivery by determining the edible plant(s) half-life, toxicity and specificity. Indeed, based on the selection of the plant(s), edible nanoparticles should be evaluated for their ability to enhance tissue/cell specificity, as in the case of GDNPs that have been reported to target different tissue and cells from grapefruit edible nanoparticles 12.The edible plant(s) that will be used may also have beneficial effects to the organism apart from being safe for consumption, as illustrated in the case of GDNPs, that have anti-inflammatory and anti-oxidant effects.

Moreover, the duration of such a treatment needs to be carefully evaluated to determine maximum efficacy without developing side effects.

Another aspect that needs to be addressed is the feasibility of a proposed treatment with edible nanoparticles in terms of production time and costs. It should be noted that, interestingly, GDNPs can be mass-produced, a feature that makes them more attractive for therapeutic use.

Finally, in order to address the complexity of a disease like obesity, it could be necessary to combine different microRNAs whose deregulation has been described in obese patients. Researchers have reported decreased plasmatic levels of let7-a, let-7c, let-7d, let-7e, let-7f, and let-7g in obese subjects. Also, decreased expression of miR-130a, miR-130b and miR-193 along with increased expression of miR-221 have been reported in adipose tissue from obese subjects, compared to lean subjects. Other studies have investigated the role of miR-33 reporting increased expression that leads to lower plasma high-density lipoprotein cholesterol (HDL-C) and impaired reverse cholesterol efflux, thus promoting atherosclerotic plaque build-up and associating miR-33 to metabolic syndrome 9.

The knowledge of deregulated microRNAs could be of utmost importance to allow the loading of a combination of several microRNAs in edible nanoparticles. In addition, the combination of different types of edible nanoparticles with complementary effects loaded with specific groups of microRNAs could lead the way to personalized treatment, a more effective patient management and a more efficient treatment.

## Figures and Tables

**Figure 1:**
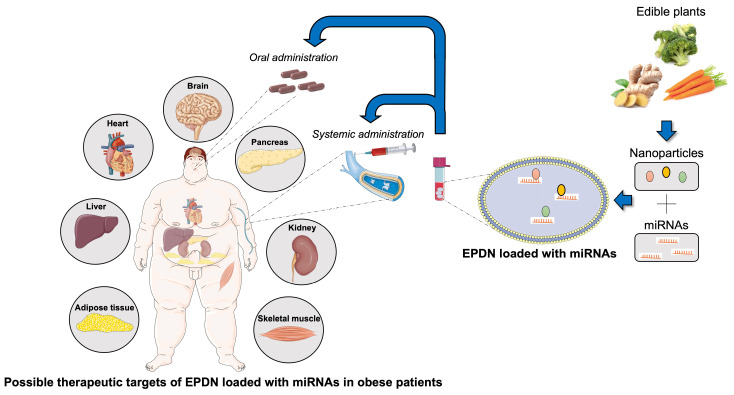
**Therapeutic potential of EPDN loaded with microRNAs in obesity.** Edible plants, such as carrots and ginger, contain a fair amount of nanoparticles that can be easily isolated. Edible plant derived nanoparticles (EDPN) represent an ideal carrier for small active molecules (e.g. microRNAs) that can be properly loaded and delivered by systemic or oral administration to target tissues on which they exert their therapeutic action.
